# Drug Resistance in Hematologic Malignancies: Induction Mechanisms, Genetics, and Therapeutics

**DOI:** 10.1155/2015/384575

**Published:** 2015-04-27

**Authors:** Fenghuang Zhan, Maurizio Zangari, Lugui Qiu

**Affiliations:** ^1^Department of Internal Medicine, The University of Iowa, Roy J. and Lucille A. Carver College of Medicine, Iowa City, IA 52242, USA; ^2^Myeloma Institute for Research and Therapy, University of Arkansas for Medical Sciences, Little Rock, AR 72205, USA; ^3^State Key Laboratory of Experimental Hematology, Institute of Hematology & Blood Diseases Hospital, Chinese Academy of Medical Science & Peking Union Medical College, Tianjin 300020, China

Hematopoietic malignancies are neoplastic tumors affecting the blood and lymphatic circulatory systems and are commonly referred to as liquid tumors. Malignancies of the blood and lymphatic systems are known to affect a whole host of cell types derived from lymphoid and myeloid progenitors including leukocytic and lymphatic cells and cells that make up the bone marrow microenvironment (Table 1). Due to the variety of cells affected, hematological malignancies of one tissue type can often result in complications with the other hematologic systems.

Hematological malignancies account for approximately 10% of all newly diagnosed neoplasms within the United States. Of the 150,000+ newly diagnosed cases of hematological neoplasms within the US, lymphomas account for 51% of the new cases followed by leukemia and myeloma (Table 1). A common characteristic of hematological malignancies is the presence of chromosomal translocations, a trait not typically observed in solid tumors [1]. This among other characteristics makes hematological malignancies a unique class of neoplasms resulting in a unique set of challenges for treatment and the prevention of relapse.

Science and medicine have focused intently over the last 20–30 years on developing treatment regimens that efficiently and effectively target and destroy cancer cells. Although our understanding of hematological malignancies has improved exponentially, resulting in greatly increased life expectancies and improved quality of life, our ability to abrogate tumor relapse has evaded us. One shortcoming to past and current therapies are centered on the tumor's innate ability to adapt and remain one step ahead of treatment regimens. The next generation of antiproliferative drugs offers a wide spectrum of therapeutic choices. Some of the more common therapies include tyrosine kinase inhibitors like Gleevec and histone deacetylase inhibitors like vorinostat, whereas there is a wealth of new therapeutics available as immunomodulatory drugs, monoclonal antibodies, antibody conjugates, and proteasome inhibitors like Velcade. This next generation of therapies represents a new hope in controlling hematological malignancy relapse and development of refractory disease.

A major underlying complication of hematological malignancies is the development of refractory disease upon patient relapse, resulting in a decreased life expectancy and quality of life [2, 3]. More recently, researchers have turned to novel mediators of drug resistance to try and explain the increase in refractory disease within hematological malignancies. Although researchers are focusing their attention on a wide variety of potential mechanisms of action, current research is centered on a small number of common themes. A few of the more widely studied areas today in drug resistance focus on the contributions of cancer stem cells (CSCs), inducers of drug efflux pump expression and inhibitors of apoptosis [4].

CSCs were originally documented and described in leukemia as a rare population of cells with limitless self-renewal capabilities [5]. Recently, CSCs have been identified in a growing number of solid tumors. A common feature of stem cells is their increased resistance to chemo- and radiotherapy [6, 7]. CSCs show plasticity and heterogeneity [8]. The plasticity indicates an equilibrium between a cell with a stem cell phenotype and mature terminally differentiated tumor cells, while the heterogeneity includes both intraclonal heterogeneity and distinct molecular mechanisms which are essential for tumor development and progression [8]. Unfortunately current chemotherapeutic treatments mainly focus on debulking tumor cells while CSCs escape these conventional therapies. Nevertheless, the upsurge in the development of drugs to target CSC-related pathways, such as Akt, Wnt/*β*-catenin, Notch, and Hedgehog, is showing promising preclinical and clinical results in hematological malignancies [9]. We expect that targeting CSCs will surely lead this field to evolve for overcoming drug resistance in the next few years.

Drug resistance is a universal problem with current therapies for hematologic malignancies, but very little is known about the molecular mechanisms. This special issue of* BioMed Research International* focuses on drug resistance in hematologic malignancies, induction mechanisms, genetics, and therapeutics. It is our goal that the reader leaves with an improved understanding of the underlying mechanisms of drug resistance in hematological malignancies and the discoveries focusing on drug responsiveness resulting in improved quality of life and increased life expectancy.

*Fenghuang Zhan* *Maurizio Zangari* *Lugui Qiu*

## Figures and Tables

**Table 1:** Types of hematological malignancies and rate of diagnosis for all newly diagnosed neoplasms within the United States.

Hematological malignancy	Percent of hematological malignancies	Percent of malignancy
*Lymphomas *	51%	
Hodgkin's Lymphoma		14%
Non-Hodgkin's Lymphoma		86%
*Leukemias *	33%	
Acute lymphoblastic leukemia (ALL)		11%
Acute myelogenous leukemia (AML)		36%
Chronic lymphoblastic leukemia (CLL)		30%
Chronic myelogenous leukemia (CML)		11%
Other		11%
*Myelomas *	15%	
Total		**100%**

(National Cancer Institute 2007–2011 rates http://seer.cancer.gov/ and The Leukemia and Lymphoma Society 2014 rates http://www.lls.org/).
